# Photon-counting detector computing tomography in treatment planning of head-and-neck cancer

**DOI:** 10.1016/j.phro.2026.100995

**Published:** 2026-05-10

**Authors:** Enkelejda Lamaj, Patrick Wohlfahrt, Lotte Wilke, Hatem Alkadhi, Niccolo Bertini, Hubert Gabrys, Matthias Guckenberger, Panagiotis Balermpas, Stephanie Tanadini-Lang, Serena Psoroulas

**Affiliations:** a Department of Radiation Oncology, University Hospital Zurich (USZ), University of Zurich (UZH), Zurich, Switzerland; b Siemens Healthineers, Cancer Therapy Imaging, Forchheim, Germany; c Diagnostic and Interventional Radiology, University Hospital Zurich (USZ), University of Zurich (UZH), Zurich, Switzerland; d Radiation Oncology Unit, Oncology Department, Azienda Ospedaliera Universitaria Careggi, Florence, Italy

**Keywords:** Photon-counting detectors, Computer tomography, Head and neck cancer, Metal artifact reduction

## Abstract

**Background and purpose:**

Photon-counting detector computed tomography (PCD-CT) offers several image reconstruction options, e.g. virtual monoenergetic images (VMIs) and relative electron density (RED) images. This study investigated these PCD-CT reconstructions for dose calculation in head-and-neck cancer (HNC) patients.

**Materials and methods:**

10 HNC patients referred to radiotherapy were scanned with PCD-CT. The same contouring and therapy strategy was followed as in the patients’ clinical treatment, optimized on an energy-integrated-detector CT (EID-CT). 18 volumetric arc therapy treatment plans (6 MV photons) were optimized on RED images and recalculated on 70 keV VMI (VMI70), using the same amount of monitor units. While RED images did not require scanner-specific calibration, the conversion from CT number to RED for VMI70 was calibrated using a phantom with tissue-equivalent inserts with known RED. To assess the clinical impact of using RED maps with vendor-provided calibration, one plan per patient was recalculated using bulk material overrides. Plan quality was evaluated by evaluating dose-volume metrics, such as the dose to 98% of planning target volume, and compared between RED, VMI70, and EID-CT.

**Results:**

All plans optimized on RED images fulfilled the clinical goals. Dose-volume metrics differed by <3% between RED and VMI70 and <5% between RED and EID-CT. RED differed by differed by <4% from bulk density override (<1% when the target did not overlap with air volumes).

**Conclusions:**

Treatment planning can be accurately performed on PCD-CT images of HNC cases, using either VMI (after CT-number-to-RED conversion) or RED images (without scanner-specific calibration).

## Highlights

• Plans optimized on photon-counting detector computed tomography images differ <5% from conventional plans.
• Virtual monoenergetic images (70 keV) need artifact reduction and calibration.
• Electron density reconstructions do not require clinic-specific calibration.

## Graphical abstract

**Figure:**
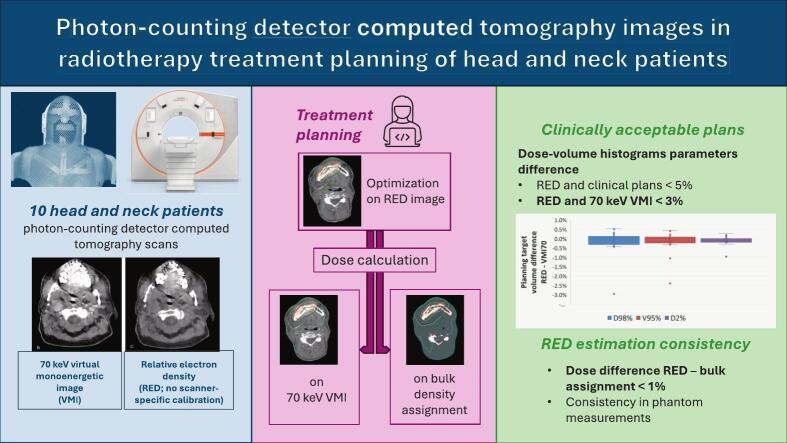

## 1 Introduction

Computed tomography (CT) imaging is a cornerstone of the radiotherapy workflow, with simulation CT scans routinely converted into relative electron density (RED) maps for accurate dose calculation, through Hounsfield units look-up tables (HLUTs). Photon-counting detector CT (PCD-CT) represents the latest advancement in CT technology, offering several advantages over conventional energy-integrating-detector CT (EID-CT), including higher spatial resolution, improved signal- and contrast-to-noise ratio, and higher radiation dose efficiency [1], [2], [3], [4], [5]. Since the detector can discriminate photons of different energy collected in a single exposure, it allows to reconstruct virtual monoenergetic images (VMI) and RED maps as weighted linear superposition of CT images reconstructed based on the low- and high-energy bin, using similar concepts as developed for dual-energy CT (DECT) scanners [6], [7].

In VMIs, the reconstruction algorithm assigns each voxel a CT number (in Hounsfield units, HU) that corresponds to the interaction cross section at the chosen energy. High-energy VMIs are less sensitive to the presence of metal implants [8], while low-energy VMIs have better soft-tissue contrast [9]. Furthermore, the RED of each voxel can be obtained from the photon interaction cross-section [10], and is independent of the tube voltage used for CT acquisition. RED images may improve dose calculation accuracy by removing the need for HLUT calibration [11] and can also be directly converted into mass density using the algorithmic principle described in Wohlfahrt, Möhler [12]. Reconstruction of VMI and RED images have been investigated with DECT [10], [13], [14], and confirmed the feasibility of using both images based on DECT with custom HLUT. PCD-CT however uses different detectors and reconstruction algorithms with respect to DECT [15], [16], and therefore it is important to validate the usage of PCD-CT-derived spectral images for quantitative tasks such as dose calculation ahead of their introduction in the clinical workflow.

We therefore investigated the usage of RED and VMI images from PCD-CT in the radiotherapy treatment planning of head-and-neck cancer (HNC) patients, where image quality is often challenged by the presence of metal implants.

## 2 Materials and methods

### 2.1 Patient cohort

The patient coht consisted of 10 HNC patients referred froutine radiotherapy. They received a diagnostic PCD-CT scan as part of their evaluation prito treatment. Additional inclusion criteria were completion of the diagnostic examination prito radiotherapy, age over 18 years, and presence of radio-opaque artificialal implants. Exclusion criteria included pregnantbreastfeeding patients. All data was collected prospectively following approval by the cantonal ethics committee (number: 2022–00676). Only data collected during the standard clinical diagnostic process was included, ensuring no additional burden on the patients. The patients’ characteristics (location of the implant with respect to the planning target volume (PTV), artifact presence) are summarized in Table 1.

**Table 1:** Patient and plan characteristics. PTV: planning target volume.

Patient number	Artifacts: location and override	Planning strategy
1	implants in oral cavity, outside of PTV	two series: 25 × 2 Gy, 5 × 2 Gy, two PTVs
2	strong artifacts within PTV (required tissue override during planning)	30 × 2 Gy, single PTV
3	implants very close to PTV, no overlap	35 × 2 Gy, single PTV
4	implants within PTV, no strong artifact	three series: 25 × 2 Gy, 5 × 2 Gy, 5 × 2 Gy, three PTVs
5	strong artifacts in mouth and lips, close to PTV (required tissue override during planning)	33 × 2.12 Gy, three PTVs, simultaneously-integrated-boost
6	strong artifacts in oral cavity, adjacent to PTV (required tissue override during planning)	30 × 2 Gy, two PTVs, simultaneously-integrated-boost
7	strong artifacts in oral cavity, within PTV (required tissue override during planning)	two series: 25 × 2 Gy, 5 × 2 Gy, two PTVs
8	implants within PTV, no strong artifact	two series: 25 × 2 Gy, 5 × 2 Gy, two PTVs
9	strong artifacts in oral cavity and lips, within PTV (required tissue override during planning)	two series: 25 × 2 Gy, 5 × 2 Gy, two PTVs
10	implants within PTV, no strong artifact	three series: 25 × 2 Gy, 5 × 2 Gy, 3 × 2 Gy, three PTVs

### 2.2 Imaging

All patients were scanned with a first-generation dual-source PCD-CT system (NAEOTOM Alpha, Siemens Healthineers, Forchheim, Germany) approved for clinical use in radiology, using a standard single-source protocol, with 120 kV tube voltage and 81 mAs exposure.

As the mean photon energy in the spectrum of a single-energy EID-CT acquired at 120 kV is close to 70 keV [17], we chose 70 keV VMI (VMI70) to test a PCD-CT workflow very similar to EID-CT. A comparison between our institute EID-CT images and the VMI70 images is reported in Supplementary Materials A (Fig. S1). We performed a HLUT calibration of the VMI70 using the CIRS 062 electron density phantom, with tissue-mimicking inserts of known electron and mass density. The calibration was performed using linear interpolation following our institute’s clinical procedure (Supplementary Materials A, Fig. S2). We inserted the resulting HLUT (for mass and relative electron density) in our clinical treatment planning system (TPS) Varian Eclipse (Siemens Healthineers, Palo Alto, CA, US) for dose calculations.

RED maps of the patient scans were reconstructed using a product development prototype software for the NAEOTOM Alpha PCD-CT scanner with software version VB20, which at the time of the study was pending CE certification for clinical use. RED maps were reconstructed as DICOM-compliant images, with voxel values, *H*, in HU defined as

(1) H=(RED-1)∗1000

RED and mass density curves (derived using the method from Wohlfahrt, Möhler [12]) were provided by Siemens Healtineers, and imported in the TPS without a scanner-specific calibration.

**Fig. 1:**
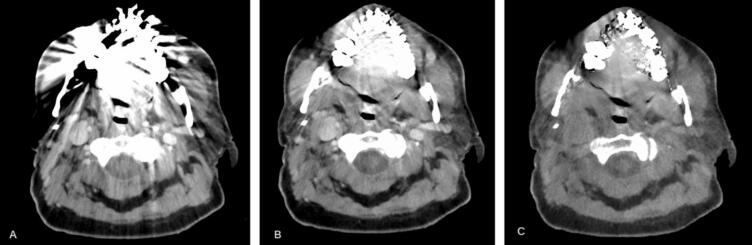
Patient 9 PCD-CT reconstructions. A) Virtual monoenergetic image of 70 keV (VMI70) without iterative metal artifact reduction (iMAR); B) VMI70 with iMAR; C) Relative electron density (RED) image with iMAR. All images shown in the abdomen window (CT values between −125 and 225 HU). (For interpretation of the references to colour in this figure legend, the reader is referred to the web version of this article.)

As all patients in our cohort had metallic implants, we used the iterative metal artifact reduction (iMAR [18], Siemens Healthineers, Forchheim, Germany) with the dental fillings option. The impact of iMAR is shown for an example patient (Patient 9, Fig. 1). Artifact correction is enhanced in the RED map (due to the influence of the high-energy bins in the RED map reconstruction).

### 2.3 Planning approach

The PTVs were delineated on the VMI70 by an experienced physician while the organs of interest (OOI) [19] were either contoured by the physician (mandible, oral cavity, parotids, pharynx, submandibular glands) or generated by an auto-contouring software (all other OOIs generated with MIM, A GE Healthcare company, Cleveland, OH, US). The patients were planned with volumetric modulated arc therapy (VMAT) in Varian Eclipse for a Varian TrueBeam (Siemens Healthineers, Palo Alto, CA, US) with 6 MV according to their original therapeutic concept, resulting in 18 plans in total (Table 1). Depending on the target location, two full arcs or two partial arcs were used. Prior to plan optimization, voxels with mass density higher than 3 g/cm^3^ were detected and overridden to titanium (mass density 4.42 g/cm^3^, relative electron density 4.107 and 3107 HU in RED map, relative electron density 3.678 and 10,487 HU in 70 keV VMI image). For 6 out of 10 patients, some of the artifacts were in the PTV or near the PTV region, and overridden to muscle skeletal tissue (relative electron density of 1.039, corresponding to a CT number of 36 HU in the VMI70 calibration, and 39 HU in the RED map). In addition, air in the PTV larger than 5 mm was detected and overridden to water for the optimization but not for final dose calculation, to prevent the formation of hotspots in the air cavities during the optimization. We used an in-house RapidPlan model [20] for optimization, editing the optimization objectives as necessary to achieve better PTV coverage and OOIs sparing. All plans were optimized on the RED images and normalized to the PTV mean dose (D_mean_) set to the prescribed dose. The calculation was performed with Acuros 16.1 [21], the same dose calculation engine used clinically, using as input the RED maps and the mass density conversion. The plans were then recalculated on VMI70 using the same number of monitor units.

### 2.4 Plan evaluation

We evaluated plan quality using different dose-volume histogram (DVH) parameters. For the PTV, we considered the minimum dose covering 98% (D_98%_) and 2% (D_2%_), and the volume covered by 95% of the dose (V_95%_). For the OOIs, we used the D_mean_ and dose to 0.1 cm^3^ volume (D_0.1cm3_). Plans were considered clinically acceptable if D_98%_>90% (quantifying coverage) and D_2%_<105% (quantifying maximum dose) for the PTV [22]. OOI dose guidance [19] followed international guidelines [22]. We calculated the differences between RED and VMI70 for each DVH parameter, and performed a non-parametric sign test to evaluate significant differences in PTV and OOI DVH parameters. We also compared the RED DVH parameters with the values from the patients’ clinical plans, optimized and calculated on EID-CT images. However, since the patients were in a different position between EID-CT and RED scans, a 3D dose comparison between the two plans was not possible.

### 2.5 Bulk calculation

To investigate how variations in the RED value (and the uncertainties in its estimation, as the true value is unknown) affect the dose distribution, we recalculated the dose on RED images with CT numbers overridden to the bulk density of the specific tissue. Specifically, we segmented on the RED image the regions corresponding to air, lung, soft tissue (including both fat and muscle tissues), and bone. High density material and possible artifact override used in the planning process was kept separated from the surrounding structures (bone and soft tissue). These structures were then assigned a specific relative electron density corresponding to the tissue definition (Table 2). The assumed densities followed the values used in our clinic for the validation of synthetic CT generation from MRI images [23], and match well the average CT number found in each structure. We then recalculated the plan with the same monitor units as in the RED plan and compared dose statistics for both PTV and OOIs. We consider dose differences between the bulk density recalculated plan and the nominal plan in the range of −4% to + 1% to be clinically acceptable. The range was defined from studies in the thorax and abdominal region at our institute [23]; the asymmetry in the range is due to a systematic difference observed in that previous study.

**Table 2:** Structures and material definition for the bulk calculation, segmented on the relative electron density image. Quantities are averaged over all ten patients considered in the study.

Structure	Image thresholding	assumed relative electron density	CT number assignment, in HU	average CT number in structure, in HU	Standard deviation CT number in structure, in HU
Air	CT number <−850 HU	0	−1000	−985	9
Bone	Eclipse bone segmentation	1.4	400	416	42
Lung	Eclipse lung segmentation, only if dose in lung region	0.25	−752	−823	51
Soft tissue (including fat and muscle)	Body excl. air, lung, bone, and potentially high density/artifact regions	1.007	7	8	7

## 3 Results

**Fig. 2:**
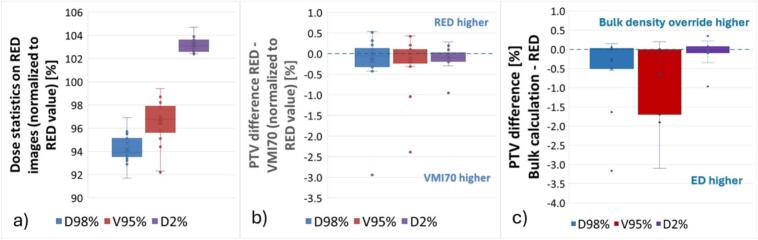
(a) PTV DVH parameters from the plans optimized on the red image. (b) The PTV DVH parameters difference between red and vmi70 (normalized to the red value). (c) The PTV DVH parameters difference between the bulk calculation and the red, normalized to the red.

**Fig. 3:**
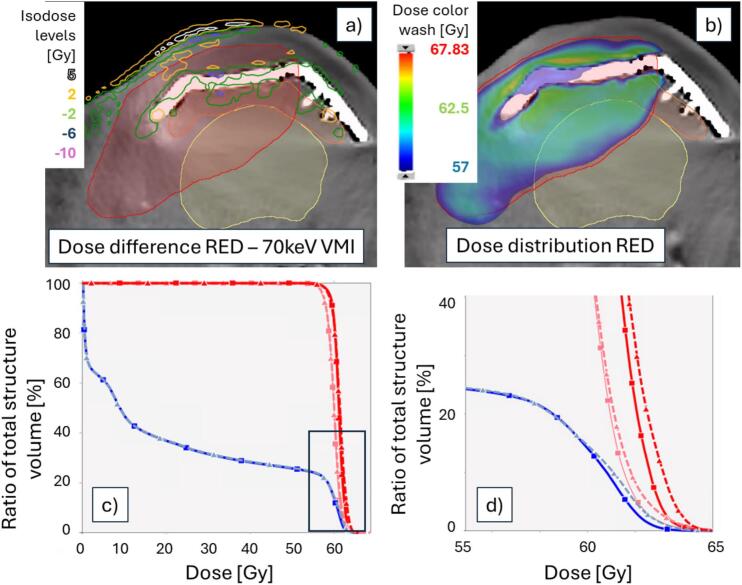
A) Example of local dose differences (red-vmi70) in the high dose region for patient 2 (prescription dose 60 Gy). The isodose lines show dose differences above 3%; large differences are concentrated around the metallic implant (a titanium reconstruction implant attached to the Mandible). The thin contours show: red, the PTV, blue, the mandible, yellow, the oral cavity. b) dose distribution in the corresponding slice on the RED image. c) DVH for the corresponding patient, showing the impact, mainly visible as a difference in the maximum dose in the mandible (blue), PTV (pink) and gross tumor volume (red). Dashed lines are VMI DVHs, solid lines RED DVHs. d) magnification of the region with the largest difference between the DVH curves. (For interpretation of the references to colour in this figure legend, the reader is referred to the web version of this article.)

All plans, optimized and calculated on RED images, were clinically acceptable. For the PTV (Fig. 2), D_98%_ was above 90% for all plans; V_95%_ was below 95% for three plans. D_2%_ was below 105% for all cases. When recalculating the plans on VMI70, small dose differences were visible. In the PTV, most differences were below 2% (normalized to the DVH parameter value on the RED image, Fig. 2). Larger differences between the two dose calculations were found on images with large artifacts in the PTV (Fig. 3). In these images, also slightly higher doses were present in VMI70. All differences in the PTV between RED and bulk RED maps were within the clinically acceptable range (−4% to +1%, Fig. 2).

When comparing mean doses for OOIs, the difference in most cases was below 1%. We also tested different clinically relevant DVH parameters for the OOIs; only Parotid R, Submandibular Gland R, Oral Cavity and Mandible D_mean_ values showed a significant difference (p-value <0.05; Fig. S4 and Table S2 in supplementary materials). No major differences were seen in OOIs in the bulk recalculations (Fig. S5 in supplementary materials).

**Fig. 4:**
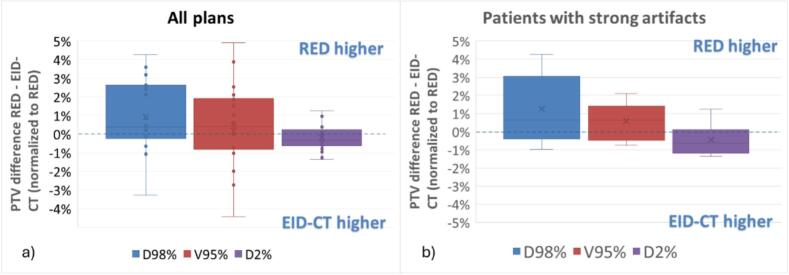
PTV DVH parameters between plans calculated on the RED image and the original treatment plan, calculated on an EID-CT scan, normalized to the RED values. On the left, the summary of all plans; on the right, the values for patients with strong artifacts.

Comparison between EID-CT plans and RED plans showed an agreement between the DVH parameters better than 5% (Fig. 4). We found that on average the coverage for RED plans was higher than for EID-CT plans. The effect was more pronounced in patients with strong image artifacts, where all plans except one showed higher coverage in the RED optimized plans (Fig. 4). The dose maximum was also slightly smaller for RED plans, but always within 1% between the two calculations.

## 4 Discussion

We created treatment plans based on PCD-CT images of HNC patients. All patients were successfully planned on RED images according to their prescribed doses. Three plans showed low coverage (V_95%_< 95%), a result we suspect is linked to the presence of a large high-density structure within the PTV. DVH parameters from plans optimized on RED images agreed within 5% with those from the clinical plan optimized on EID-CT, with a tendency for better coverage on RED images, more pronounced in patients with strong imaging artefacts. A more quantitative comparison between the 3D dose distributions was however not possible, due to the different positioning of the patient in the two scans. Since RED images are not yet available in all PCD-CT scanners worldwide, we also investigated the usage of VMIs for treatment planning, using 70 keV VMI as it provides image impression comparable to a conventional 120 kVp EID-CT [17]. We obtained very consistent results between the plans optimized on RED images and VMI70 dose recalculations, therefore, a treatment planning workflow based on VMIs (after HLUT calibration) would be also clinically feasible. A significant difference (p < 0.05) between RED and VMI70 calculations was observed only for the mean doses of the parotid, submandibular gland, mandible and oral cavity. Since all patients in our cohort had metal implants in the mouth, and the presence of artifacts correlates with a higher dose, we can preliminarily conclude that using VMIs might lead to an overestimate of the dose with respect to RED-based calculations.

We used a recalculation on bulk density assignments as a way to assess the clinical impact of the RED value assignment without a scanner-specific calibration. The dose recalculation showed minimal differences; in only three patients the PTV V_95%_ and D_98%_ difference exceeded the 1% level, but was still within clinical tolerances. In parallel, we also validated RED values based on phantom measurements (Table S1 in supplementary materials).

Our study was limited to only 10 patients (18 plans) and two image reconstructions. However, we considered all concepts used in our clinical practice (subsequent series for primary tumor and elective nodes irradiation, and simultaneously integrated boost), and therefore we do not expect any applicability issues in a wider patient population of HNC patients. We always used the Qr40 kernel for dose calculation; this is the recommended kernel for dose calculation, however, depending on the patient population, different kernels or sharpness may be used. Concerning VMIs, provided that MAR is applied and an HLUT is used, our findings would be equally applicable to other VMI energies. MAR is necessary for low VMI energies [8], [10], [24], and in some cases was shown to be important also at higher VMIs [18], [25]. From our experience, we recommend MAR in presence of dental implants also for RED images.

A key advantage of RED images is that they require only one single scanner- and protocol-independent CT conversion curve in the treatment planning system, valid also in case different tube voltages are used in the protocols [26]. Spectral imaging such as DECT can also provide RED images [27]. Our study shows that RED images from PCD-CT scans can be used in the radiotherapy planning workflow. Recent work from other groups, albeit in different anatomical regions and investigating contrast agent suppression, also show similar results [28]. However, one important limitation of our study was that we considered only PCD-CT images, mainly due to practical reasons (limiting the number of scans to the patients and scanner availability). Phantoms studies showed comparable performance in material separation between PCD-CT and DECT [29] but potentially less sensitivity to positional artifacts in PCD-CT over DECT [30]. A full comparison of PCD-CT features vs. DECT in terms of dose calculation in clinical cases is an important question, which we could not investigate in this study.

Our results show that RED maps and VMIs obtained from PCD-CT scans can be successfully used in clinical practice; in the case of RED images, without any scanner-specific calibration. Therefore, PCD-CT images can be utilized in the radiotherapy workflow, opening the possibility to exploit their potential on image quality.

## CRediT authorship contribution statement

**Enkelejda Lamaj:** Writing – review & editing, Writing – original draft, Methodology, Investigation, Data curation, Conceptualization. **Patrick Wohlfahrt:** Writing – review & editing, Software, Data curation. **Lotte Wilke:** Writing – review & editing, Data curation. **Hatem Alkadhi:** Writing – review & editing, Supervision, Resources. **Niccolo Bertini:** Writing – review & editing, Data curation. **Hubert Gabrys:** Writing – review & editing, Data curation. **Matthias Guckenberger:** Writing – review & editing, Supervision, Resources. **Panagiotis Balermpas:** Writing – review & editing, Supervision, Resources, Project administration, Funding acquisition. **Stephanie Tanadini-Lang:** Writing – review & editing, Writing – original draft, Supervision, Project administration, Funding acquisition, Conceptualization. **Serena Psoroulas:** Writing – review & editing, Writing – original draft, Visualization, Supervision, Methodology, Investigation, Formal analysis, Data curation, Conceptualization.

## Declaration of competing interest

The authors declare the following financial interests/personal relationships which may be considered as potential competing interests: The department of radiation oncology, Zurich University Hospital, has a research agreement on photon counting CT with Siemens Healthineers. Patrick Wohlfahrt is an employee within the research and development team of Siemens Healthineers. The Department of Diagnostic and Interventional Radiology has research agreements with Bayer, Canon, Guerbet and Siemens. H.A. received speaker’s fee from Siemens.
