# Supplementary material for: Photon-counting detector computing tomography in treatment planning of head-and-neck cancer
Source: Phys Imaging Radiat Oncol. 2026 May 10;39:100995. doi: 10.1016/j.phro.2026.100995 (PMC13196142; doi:10.1016/j.phro.2026.100995)
Supplement: Supplementary data 1 [file mmc1.pdf]

Photon-counting detector computing tomography in treatment planning of head-and-neck cancer – supplementary materials

Supplementary material A: Comparison of photon-counting detector (PCD) and energy-integrating detector (EID) CT.

For illustration purposes, we report images from the same patient, from the simulation energy-integrating detector CT (EID-CT, Figure S1A) and the photon-counting detector CT (PCD-CT, Figure S1 B, C). The simulation CT was performed 10 days after the PCD-CT scan. The patient position was different between the two images therefore no direct, voxel-to-voxel comparison of CT-numbers can be performed. The images are shown at a similar slice location (location of a mandible implant), to highlight differences in the artifact reduction. In this study, we used the Qr40 kernel. The acronym stands for ‘Quantitative regular', a reconstruction kernel with an accurate definition of CT numbers without additional visual edge enhancement. This is appropriate for tasks requiring high accuracy and precision such as dose calculation. The '40' refers to the sharpness of the image reconstruction, representing an intermediate resolution (“soft” image impression), which is especially suited for representation of soft tissues.

Figure S1A: a 120 kVp simulation CT scan, exposure 375 mAs, current 450 mA, performed on a conventional single-energy CT system (SOMATOM Definition AS, Siemens Healthineers). The image is reconstructed using the B30f kernel.
Figure S1B: Virtual monoenergetic image of 70 keV derived from PCD-CT, 120 kVp single-source, exposure 81 mAs, current 160 mA, performed on the NAEOTOM Alpha (Siemens Healthineers). The image is reconstructed using the Qr40 kernel.
Figure S1C: Relative electron densities (RED) image derived from the same scan as figure S1B. The image is reconstructed using the Qr40 kernel.

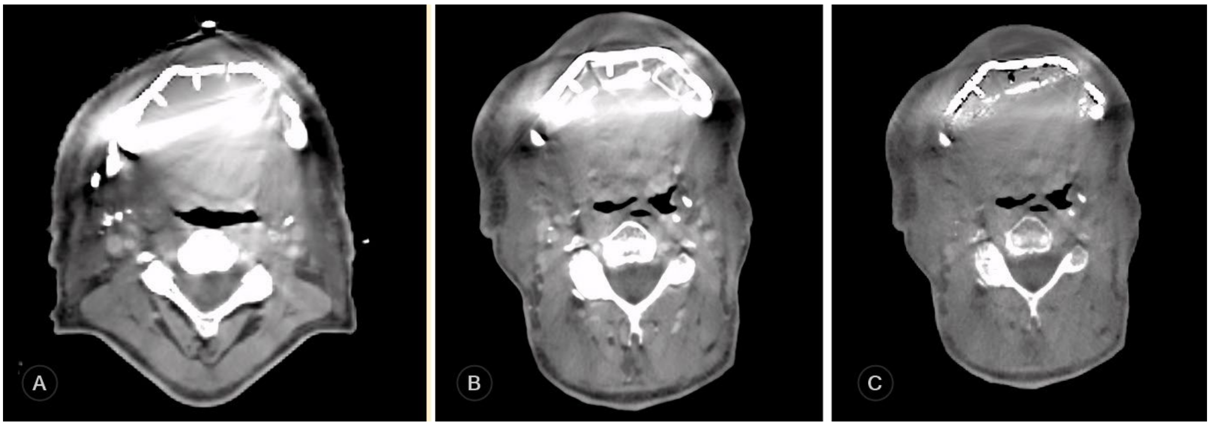

Figure S1: Images of one patient with head-and-neck cancer (patient 2) with A) EID-CT; B) 70 keV VMI from PCD-CT; C) RED from PCD-CT. All images are processed with iterative metal artefact reduction (iMAR) and shown in soft tissue window (-125 to 225 HU). The patient position is different between simulation EID-CT and PCD-CT.

For illustration we also report the calibration curve of the PCD-CT 70 keV VMI (VMI70) used in this work (reconstructed using the Qr40 kernel). The CIRS 062 electron density phantom was used for the calibration. The points in Figure S2 are measurements of different inserts, averaged over different positions inside the phantom. The list of samples and their RED is shown in Table S4. For comparison, we are also reporting the EID-CT calibration curve (120 kVp) obtained with the same phantom at the scanner used in our institute (images reconstructed with the Br38 kernel).
The last measurement point is taken at about 1000 HU (Dense bone insert). Above this point, the curve is linearly extrapolated. To avoid too large uncertainties from the extrapolation, in the dose reconstruction we capped all voxels corresponding to densities higher than titanium to the density of titanium.

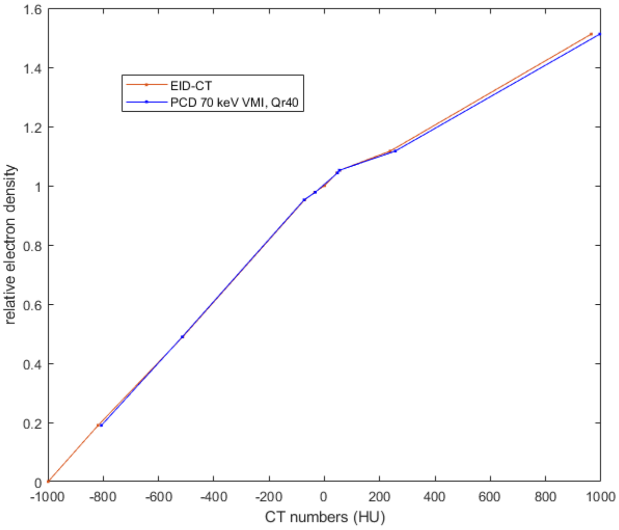

Figure S2: Calibration curve for the 70 keV virtual monoenergetic image (VMI) reconstruction (Qr40 kernel), derived from a PCD-CT phantom scan, with relative electron density measured using a phantom with inserts of known relative electron density and mass density. For comparison, the curve is overlaid with the one of the EID-CT scanner currently in use for planning CT at our institute, reconstructed with the Br38 kernel.

Supplementary material B: Validation of RED values with CIRS electron density phantom.

Table S1: Comparison of the nominal RED values for the CIRS 062 electron density phantom and the RED measured in the PCD-CT images (Qr40 kernel). RED is estimated from the mean CT number measured in the RED images, using Eq. 1 (section 2.2 of the manuscript). The 'outer insert' values are taken from the outer ring, while the 'inner insert' values are taken from the internal section, in the measurement setup shown in Figure S4. The images were scanned using a thorax radiology protocol with 120 kVp.

CIRS phantom 062		outer insert values				inner insert values			
**Material**	**nominal RED**	**mean CT number [HU] in structure**	**Standard deviation CT number [HU]**	**calculated RED**	**Difference RED (nominal - measured) relative to nominal [%]**	**mean CT number [HU] in structure**	**Standard deviation CT number [HU]**	**calculated RED**	**Difference RED (nominal - measured) relative to nominal [%]**
Air (exterior)		-998	14						
Lung inhale	0.190	-815	25	0.19	2.6	-808	19	0.19	-1.1
Lung exhale	0.489	-526	26	0.47	3.1	-517	26	0.48	1.2
Adipose	0.949	-49	25	0.95	-0.2	-51	28	0.95	0.0
Breast	0.976	-18	19	0.98	-0.6	-23	28	0.98	-0.1
Solid water	1.000	9	15	1.01	-0.9	-1	22	1.00	0.1
Muscle	1.043	46	21	1.05	-0.3	44	26	1.04	-0.1
Liver	1.053	53	19	1.05	0.0	51	19	1.05	0.2
Trabecular bone	1.117	124	24	1.12	-0.6	124	28	1.12	-0.6
Dense bone	1.456	551	23	1.55	-6.5	542	29	1.54	-5.9

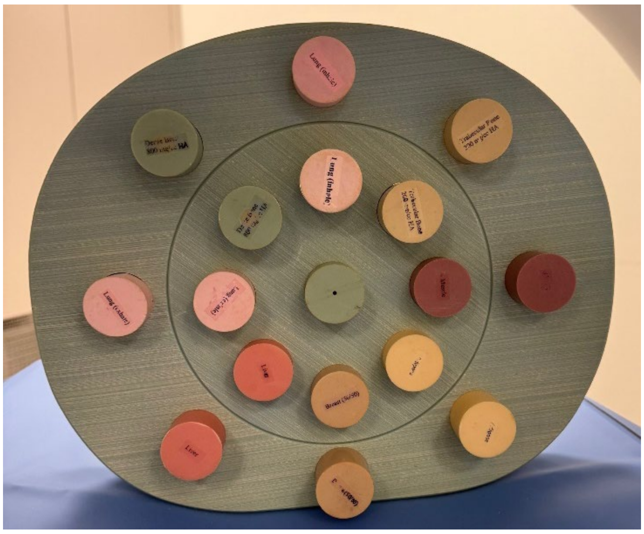

Figure S3: Phantom setup for the RED measurements reported in table S3. The 'outer insert' values are taken from the inserts in the outer ring, while the 'inner insert' values are taken from the internal section. All values are estimated by measuring the average CT number in a cylindrical structure fully inside the tissue insert.

Supplementary material C: Dose statistics comparison for organs at risk (OAR).

As part of the comparison between RED and VMI70, we compared different clinically-relevant dose-volume histogram (DVH) parameters for all OARs. In some cases, and in agreement with our clinical practice, one DVH parameter is evaluated, in other cases more than one. We also performed a non-parametric sign test to evaluate whether the distribution of the difference for each OAR parameter was compatible with a median value of 0 (indicating equivalence between the two dose distributions).

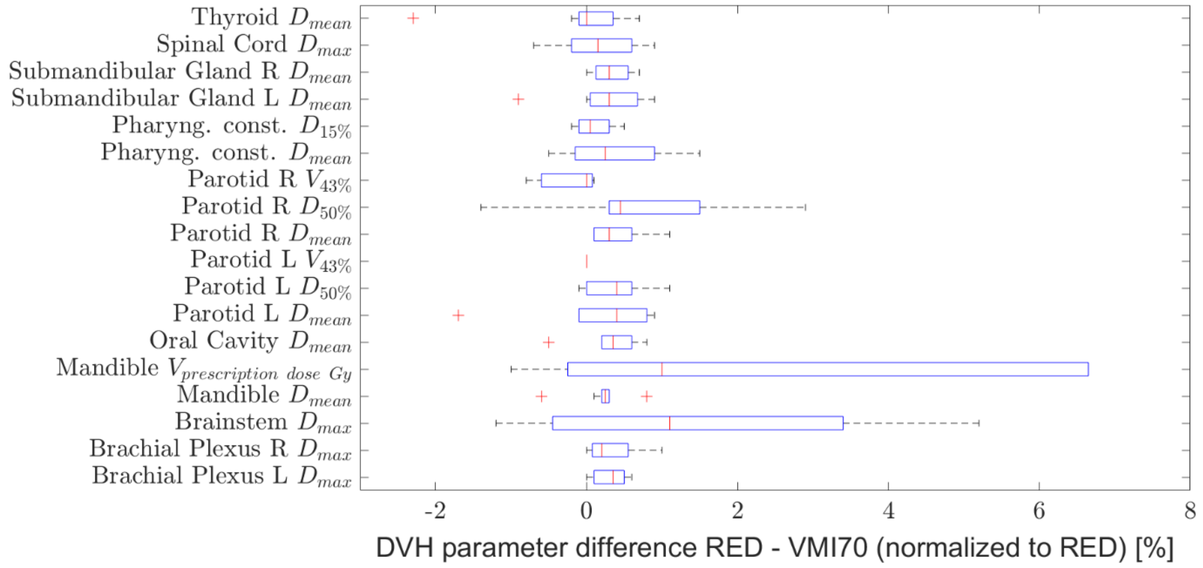

Figure S4: Difference of the DVH parameters used clinically to evaluate the risk of toxicity in each OAR, between the dose calculated on the RED image and on the 70 keV virtual monoenergetic image (VMI70), for all patients in the cohort. The difference is normalized to the RED values.

Table S2: Results of the sign test between dose values on RED and on VMI70 for all OARs and all DVH parameters considered per each OAR in the study. We considered a p-value < 0.05 significant (values reported in bold).

Structure	***p-value***
Brachial Plexus L D_{max}	0.22
Brachial Plexus R D_{max}	0.063
Brainstem D_{max}	0.727
**Mandible D_{mean}**	**0.022**
Mandible $V_{prescription\ dose\ [Gy]}$	0.375
**Oral Cavity D_{mean}**	**0.022**
Parotid L D_{mean}	0.754
Parotid L $D_{50\%}$	0.219
**Parotid R D_{mean}**	**0.022**
Parotid R $D_{50\%}$	0.219
Pharynx Const D_{mean}	0.625
Pharynx Const $D_{15\%}$	0.625
Submandibular Gland L D_{mean}	0.125

**Submandibular Gland R D_{mean}**	**0.016**
Spinal Cord D_{max}	0.727
Thyroid D_{mean}	0.289

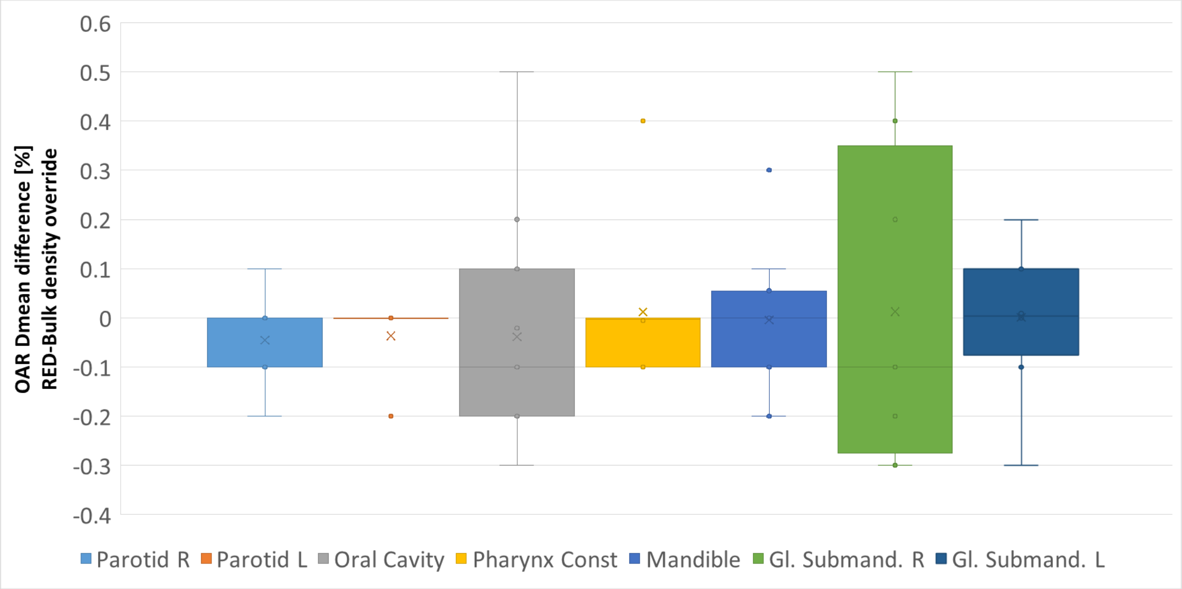

Figure S5: Difference in mean dose (as percentage of the prescription dose) for the main organs at risk between the dose calculation on RED map derived from PCD-CT and on bulk RED maps, for all patients in the cohort. The percentages are with respect to the RED values.
